# Small but Mighty—The Emerging Role of snoRNAs in Hematological Malignancies

**DOI:** 10.3390/ncrna7040068

**Published:** 2021-10-26

**Authors:** Jaime Calvo Sánchez, Marcel Köhn

**Affiliations:** Junior Research Group ‘RBPs and ncRNAs in Human Diseases’, Medical Faculty, Martin-Luther-University Halle-Wittenberg, 06120 Halle, Saale, Germany; jaime.calvo-sanchez@medizin.uni-halle.de

**Keywords:** snoRNA, ncRNA, leukemia, ribosome function

## Abstract

Over recent years, the long known class of small nucleolar RNAs (snoRNAs) have gained interest among the scientific community, especially in the clinical context. The main molecular role of this interesting family of non-coding RNAs is to serve as scaffolding RNAs to mediate site-specific RNA modification of ribosomal RNAs (rRNAs) and small nuclear RNAs (snRNAs). With the development of new sequencing techniques and sophisticated analysis pipelines, new members of the snoRNA family were identified and global expression patterns in disease backgrounds could be determined. We will herein shed light on the current research progress in snoRNA biology and their clinical role by influencing disease outcome in hematological diseases. Astonishingly, in recent studies snoRNAs emerged as potent biomarkers in a variety of these clinical setups, which is also highlighted by the frequent deregulation of snoRNA levels in the hema-oncological context. However, research is only starting to reveal how snoRNAs might influence cellular functions and the connected disease hallmarks in hematological malignancies.

## 1. The Biogenesis and Lifecycle of Small Nucleolar RNAs (snoRNAs)

It has become increasingly evident that non-protein coding RNAs (ncRNAs) play a fundamental role in regulating cellular processes, both in developmental and pathological contexts. Accounting for more than 90% of the transcribed RNAs, it has prompted researchers to develop more advanced sequencing technologies, leading to the global detection of several ncRNA classes, such as microRNAs (miRNAs), long ncRNAs (lncRNAs) and small nucleolar RNAs (snoRNAs), among others [1]. The existence of snoRNAs was proven already in the 1960s (reviewed in: [2]) but was characterised in further detail with the development of more sophisticated experimental procedures in the 1980s and 1990s [3,4,5]. SnoRNAs were considered important for fundamental cellular processes but have also been seen as unglamorous transcriptional products. However, recent evidence has demonstrated that aberrant expression of snoRNAs might play crucial roles in controlling oncogenic cell properties, especially in hematologic tumours. However, its precise contribution in the regulation of normal and malignant hematopoiesis remains largely unknown.

SnoRNAs are essential, short (60–300 nucleotides) and non-polyadenylated ncRNAs that predominantly reside in the nucleolus of eukaryotic cells and their localization implies a direct connection to their canonical function: guidance of post-transcriptional modifications and subsequent maturation of ribosomal RNAs (rRNAs) and small nuclear RNAs (snRNAs) [6,7,8]. These post-transcriptional modifications in rRNAs are essential as they are required to facilitate rRNA processing and secondary structure formation in order to accurately and efficiently produce the ribosomal machinery [9,10].

### 1.1. Classification of snoRNAs

**Figure 1:**
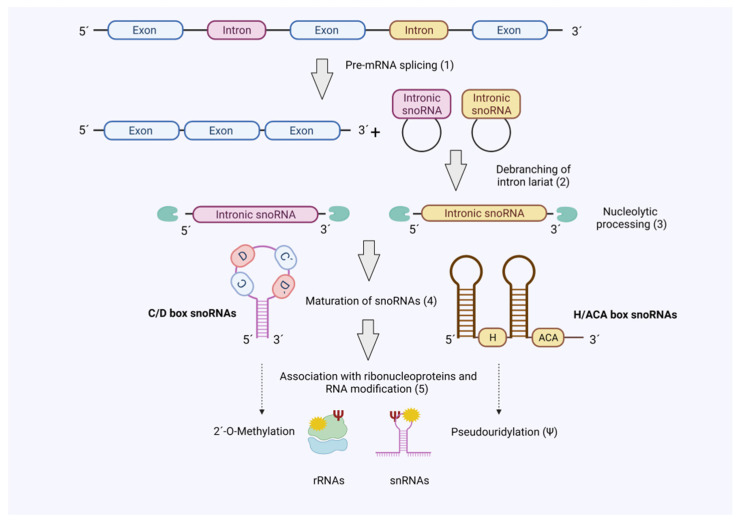
SnoRNA biogenesis. The majority of snoRNAs are produced as ‘byproduct’ during pre-mRNA splicing. Hence, mature snoRNAs associate with RNA-binding proteins in snoRNPs to guide 2′-O-methylation (C/D Box) or pseudouridylation (H/ACA Box) of target RNAs (rRNA and snRNAs). Figure created with BioRender.com (accessed on 20 October 2021).

The majority of snoRNAs are divided into two major structural classes depending on the type of evolutionary conserved sequences defined as ‘boxes’: C/D box and H/ACA box snoRNAs. These boxes are crucial as they influence overall snoRNA structure and define the binding specificity with proteins to form stable and catalytically small nucleolar ribonucleoprotein (snoRNP) complexes (Figure 1). In both cases, the snoRNAs hybridize to the relevant target RNAs (e.g., rRNA) and guide the associated protein complexes for modification of the substrate RNAs [11,12]. C/D box snoRNA structure consists of a closed loop with two conserved motifs, RUGAUGA in box C and CUGA in box D, located at the 5′ and 3′ ends of the RNA molecule, respectively. These snoRNAs form the snoRNP complex with SNU13, NOP56, NOP58 and fibrillarin (FBL), which possesses the catalytic activity for 2′-O-ribose methylation (2′-O-Me) of rRNAs [7]. This modification protects RNA from hydrolysis and modifies flexibility of the RNA molecule, which contributes to the translation capacity of the ribosomes [13]. On the other hand, H/ACA box snoRNAs consist of two stem loops connected by a hinge region or box H (ANANNA motif) with the box ACA located at the 3′ end. This snoRNA class forms the snoRNP complex with GAR1, NHP2, NOP10 and dyskerin (DKC1), the latter one endowed with the catalytic activity to carry out pseudouridylation of small nuclear RNAs (snRNAs) and rRNAs [14,15]. These pseudouridines (Ψ) have been found to provide greater rigidity to the RNA molecule by increasing duplex stability and affecting secondary structures. Interestingly, most of these Ψ are concentrated in crucial regions of the rRNAs (i.e., decoding site, mRNA channel or tRNA binding site) for the correct assembly of the ribosome and proper protein synthesis [16,17,18,19].

Additionally, small Cajal body-associated RNAs (scaRNAs) represent a subdivision from the canonical snoRNAs which accumulate in other subnuclear structures (Cajal bodies) and whose structure differs from C/D and H/ACA box snoRNAs, only including Cajal body localization signals. Although scaRNAs are involved in the same functions as the other snoRNAs, they differ in their target RNAs, which are the spliceosomal RNAs U1-U5, U12, U4atac and U6atac [20,21].

### 1.2. Biogenesis of snoRNAs

Most snoRNAs genes are encoded in introns and transcribed by RNA polymerase II (Pol II) together with their host gene. After transcription, introns from snoRNA host genes are removed by splicing. However, in contrast to classical introns snoRNA-encoding introns are subjected to a defined processing mechanism, which involves debranching of the intron, exonucleolytic processing steps and association of ribonucleoproteins that guide nucleolar localisation (Figure 1) [22,23].

As mentioned above, most of the so-far identified human snoRNAs are processed from intronic regions of either protein-coding genes or long non-coding RNAs whose function is still poorly understood. Interestingly, most of the protein-coding host genes (e.g., ribosomal proteins) encode for proteins involved in ribosome biogenesis and translation factors, thus suggesting that co-regulation of these proteins and the ‘hosted’ snoRNAs is required for efficient translation [24]. However, snoRNA expression does not always correlate with expression of the host genes. Several transcribed human snoRNA host genes produce RNA isoforms that are eventually degraded via nonsense-mediated RNA decay (NMD) [25]. This suggests that at least some non-coding host genes primarily serve a role as snoRNA-precursor.

### 1.3. Detection, Analysis and Targeting of snoRNAs

SnoRNAs represent a heterogenous class of ncRNAs regarding their structure, type of RNA modifications, targeted sites and protein interactors, thus sophisticated and interdisciplinary approaches to analyse their cellular role are required. For standard small scale snoRNA expression profiling and detection of quantitative PCR (qPCR), RNAse protection assay and Northern blotting are used. However, these methods might fail to discriminate between closely related snoRNA family members due to cross hybridisation. For these reasons, aforementioned methods are currently used as validation of large scale transcription profiling [26]. The identification and mapping of snoRNAs in the genome have traditionally been performed by microarray analysis or standard RNA sequencing (RNA-seq). Although RNA-seq has transformed the field of RNA biology allowing unprecedented mapping of the transcriptome, most next-generation sequencing approaches are focusing on profiling medium and long RNAs (>200 nucleotides) or very short sequences (17–26 nucleotides), thus snoRNA expression data is usually underrepresented in these studies. To address this gap, recent studies showed an enhanced next-generation sequencing approach with optimised library preparation and bioinformatic tools, which enables more sensitivity towards detecting novel snoRNAs, more efficient resolution of homologous snoRNA species and the capacity to discriminate between primary transcripts, mature and fully processed snoRNAs [27,28]. Furthermore, there are bioinformatic tools for prediction of RNA targets of snoRNAs available, such as snoTARGET [29], RNA snoop [30] and PLEXY [31]. However, these tools generally suggest snoRNA targets assuming that snoRNAs recognize canonical folding structures or validated snoRNA:rRNA pairs, which might not cover all snoRNA:target interactions [32]. To experimentally determine the global snoRNA target spectrum in a given cell context crosslinking and immunoprecipitation (CLIP) of snoRNP components or RiboMeth sequencing [33] as well as crosslinking, ligation, and sequencing of hybrids (CLASH, [34]) was successfully employed. To investigate the role of snoRNAs in cell depletion strategies had to be established. Since siRNA-mediated knockdowns were predicted to fail depleting the nuclear snoRNA pool, RNAse H-mediated knockdown strategies (ASOs/GapmeRs, [35,36]) or CRISPR/Cas9 techniques for snoRNA knockout/mutation [37] were used successfully.

## 2. The Role of snoRNAs in Regulating Normal and Malignant Hematopoiesis

Hematopoiesis is the life-long process by which a small population of hematopoietic stem cells (HSCs) primarily residing in the bone marrow (BM) continually gives rise to all specialised blood cell types. In adults, these HSCs are defined by their ability of self-renewal and produce multipotent progenitor cells that differentiate and produce mature blood cells [38]. These lineage-restricted progenitors (myeloid and lymphoid) ensure replenishment of the bulk of hematopoietic cells while the primitive HSCs remain in a quiescent state, protecting them from genotoxic disturbances [39,40]. Hence, in order to guarantee hematopoietic homeostasis throughout the lifetime of the organism, the balance between self-renewal and differentiation must be tightly regulated and responsive to the BM microenvironment. Insufficient differentiation along with an aberrant self-renewal capacity of the HSC pool can eventually contribute to the development of myeloproliferative diseases and leukemia [41]. HSC activity is regulated through a highly sophisticated network controlled by numerous cell-intrinsic factors involved in epigenetic, metabolic, post-transcriptional and translational processes. In particular, regulation of ribosome function has been shown to be a key factor directly linked to HSC expansion and differentiation capacity [42]. A recent study showed that the most primitive HSCs exhibited low global translation rates, but high translation efficiency of specific mRNAs required for HSC maintenance whereas myeloid-committed progenitors showed overall higher translation rates [43]. In this regard, rRNA and tRNA species are important players controlling the balance of protein synthesis and translational fidelity in HSCs. Furthermore, chemical modifications within these RNA molecules e.g., by the snoRNA-guided mechanisms, might be crucial in linking the undifferentiated state of a cell to its translational outcome in order to adapt to certain stress conditions produced in the BM environment [44].

HSCs depend on low protein synthesis to maintain an elevated proteome quality by avoiding the production of misfolded proteins. These defects can lead to a potential impairment of the self-renewal capacity of HSCs through accumulation of the well-known oncogene c-Myc [45]. It is also interesting to note that c-Myc was reported to be a master regulator of snoRNA biogenesis through direct binding to the promoter of their host genes [46]. It is readily apparent then to assume that altered protein synthesis in HSCs can disrupt normal hematopoiesis and promote the development and progression of hematological malignancies. For example, the function of the transcription factor RUNX1/AML1, frequently mutated in myelodysplastic syndrome and human leukemia, is directly linked to the expression of ribosomal proteins and rRNAs [47]. Early mutations in this gene can create pre-leukemic stem cells that through clonal growth can expand in the bone marrow and outcompete the normal hematopoietic stem and progenitor cells (HSPCs). Intriguingly, a recent study investigated a multi-step pathway involving the fusion protein AML1-ETO leading to an upregulation of certain snoRNAs, which were required for proper protein translation in leukemic stem cells and maintenance of the disease [48].

Although recent evidence suggests that snoRNAs can take part in a crucial axis along the hematopoietic process, what connects snoRNA expression and manifestation of myeloproliferative diseases remains elusive. Herein, we review results of current studies showing the prognostic potential, molecular role and therapeutic relevance of snoRNAs in different hematological tumours.

### 2.1. Acute Leukemia: Focus on Acute Myeloid Leukemia (AML)

Acute leukemia represents a useful model system to study how aberrant snoRNA expression profiles can influence leukemogenesis as this group of diseases is characterised by restrictive mutations occurring along the hematopoiesis in myeloid and lymphoid progenitors, which eventually leads to a block of differentiation. Furthermore, chromosomal abnormalities such as translocations forming oncogenic fusion proteins, i.e., AML1-ETO and MLL-AF9 in AML as well as ETV6-RUNX1 and TCF3-PBX1 in ALL, are frequently observed [49,50]. Thus, it is worth investigating if certain snoRNA signatures could be specific to clusters of pre-leukemic cells or subsets of chimeric proteins in leukemia.

Using microarrays and a high-throughput qPCR approach, Valleron et al. screened for certain snoRNA patterns specific to AML and ALL patients when compared to CD34+ progenitors, CD33+ myeloid cells (for AML patients), CD3+ and CD19+ lymphoid cells (for ALL patients) as well as healthy donors. They showed that 61% of the tested snoRNAs were downregulated when compared with their non-neoplastic counterparts [51]. Even though most of the snoRNAs appeared to be downregulated, the study showed that a cluster of snoRNAs located at the *DLK1-DIO3* locus was overexpressed in samples of acute promyelocytic leukemia (APL) carrying the PML-RARalpha_bcr1 translocation. This chromosomal region contains a subset of intronic orphan snoRNAs (SNORD112, SNORD113 and SNORD114) produced from the Meg8 transcript whose function is still unknown. One snoRNA variant, the SNORD114-1 (14q(II-1)), was experimentally validated to have an impact on leukemic cell growth via inhibiting cell cycle and the retinoblastoma (Rb) pathway. Intriguingly, only 40% of the snoRNA variants are expressed in APL patients and the underlying regulatory mechanism could not be determined. However, the fusion protein PML-RARalpha seemed to have a role in promoting snoRNA expression as binding sites for PML-RARalpha were reported close to the *DLK1* gene [52]. Interestingly, the expression of these snoRNAs was lost under all-trans retinoic acid-induced (ATRA) differentiation but increased when PML-RARalpha was re-expressed in PML-RARalpha-negative cell lines. This is consistent with other reports showing high expression of ncRNAs located in the *DLK1-DIO3* locus in hematopoietic and progenitor cells [53,54]. Continuing this line of studies, it was reported that expression of SNORD113-3, SNORD113-4, SNORD114-2 and SNORD114-3 was significantly higher in APL when compared to other types of hematologic tumours including AML, multiple myeloma and large B-cell lymphoma. Interestingly, these snoRNAs are transcribed from the 14q32 region, which is known to be associated with the development of myeloproliferative diseases [55]. In accordance, SNORD114-3 expression was reported to be highly correlated with t(15;17), the genetic hallmark translocation in APL. The authors suggested that this snoRNA could be used as a potential prognostic marker to differentiate between APL-negative and APL-positive cases among both pediatric and adult AML [56].

To precisely and comprehensively screen for global snoRNA expression, Warner and colleagues developed an optimised next-generation sequencing method with a special focus on hematopoiesis and AML [27]. The study showed that snoRNAs are the most abundant sncRNAs among all the hematopoietic subpopulations tested. They demonstrated that certain clusters of orphan snoRNAs exhibit lineage-specific expression patterns, especially those associated with the imprinted *DLK1-DIO3* and *SNURF/SNRPN* loci. The *DLK1-DIO3* locus was reported to contain 47 orphan C/D box snoRNAs with high expression levels in CD34+ progenitor cells, which dramatically decreased during granulocytic differentiation. The *SNURF/SNRPN* loci contained two C/D box snoRNA clusters, SNORD115 and SNORD116, showing a similar expression pattern. Interestingly, they also showed differential expression of several snoRNAs targeting the peptidyl transferase center (PTC) and the intersubunit bridge (ISB) of the ribosome. SCARNA15, which mediates the pseudouridylation of the U2 spliceosomal RNA [57], was decreased 2.8-fold in AML samples when compared to CD34+ cells. SNORA21 and SNORA36C, which modify the PTC and ISB respectively, were downregulated as well [27]. The authors also highlighted that reduced expression of SNORA21 was correlated with specific AML subtypes harbouring genetic mutations in the spliceosomal machinery. Genomic deletion of SNORA21 in K562 cells (erythroleukemia) impaired normal ribosome biogenesis and reduced the global translation rate. Overall, they observed a decreased expression of snoRNAs targeting site-specific modifications of the PTC and ISB of the 60S ribosome in AML samples compared to hematopoietic stem/progenitor cells [58].

The snoRNA mediated RNA modification on rRNAs, 2′-O-methylation, is the most abundant rRNA modification and has also been recently shown to be of high relevance in promoting leukemogenesis. SNORD42A was reported to be highly expressed in primary AML blasts when compared to CD34+ progenitors, monocytes and granulocytes from healthy donors [59]. This C/D box snoRNA directs site-specific 2′-O-methylation of 18S ribosomal RNA and functional knockout of SNORD42A decreased 18S-U116 methylation, impaired colony formation potential and inhibited proliferation of AML cell lines. The authors argue that this site-specific methylation is required for the conformational switch of the ribosome and ultimately influences overall protein translation rates.

Interestingly, SNORD42A was reported, among other C/D box snoRNAs, such as SNORD15, SNORD47, SNORD52, SNORD58, SNORD104, to be bound by nucleophosmin 1 (NPM1), a well-known phosphoprotein residing in nucleoli [60,61]. The *NPM1* gene bears frequent mutations in hematological diseases, especially in AML, with approximately 30% of patients harbouring a frameshift mutation resulting in aberrant NPM1 cytoplasmic localisation (NPMc+) [62]. The aforementioned study showed that snoRNAs were the most abundant RNA species bound to NPM1. Functional knockout of NPM1 resulted in a significant reduction of five 2′-O-Me modifications in 28S rRNA in mouse embryonic fibroblasts [61]. Even though 2′-O-Me levels were compromised, global translation rates were not altered. In contrast, specific protein changes were observed for Cdkn1b, Xiap, Vegf and Fgf2 with no alteration on the transcript level, suggesting translational effects. The reduction in 2′-O-Me levels was also seen in human AML samples and AML cell lines having the NPMc+ mutation, while the overall abundance of snoRNAs was not changed. Inactivation of SNORD15, SNORD47 and SNORD104 led to a decreased colony formation potential while knockout of SNORD15, SNORD52 and SNORD58 promoted erythroid differentiation in K562 cells. Together, the importance of NPM1-mediated translation regulation through direct binding of C/D box snoRNAs and its relevance in regulating cellular growth, differentiation and hematopoietic stem cell maintenance was highlighted [61].

Following this line of evidence, C/D box snoRNAs were also analysed in the AML context driven by the fusion protein AML1-ETO. The authors identified a pathway where AML1-ETO required the presence of the groucho-related amino-terminal enhancer of split (AES) in AML [48]. AES was capable of inducing snoRNP complex formation via the RNA helicase DDX21 to eventually maintain the self-renewal capacity of leukemic stem cells. Genomic deletion of either SNORD14D or SNORD35A suppressed clonogenic potential of leukemia cells *in vitro* and significantly delayed leukemogenesis in immunodeficient mice. Reduced expression of SNORD34 and SNORD43 was able to impair clonogenic growth of AML cells. Furthermore, it was demonstrated that other well-known AML oncoproteins, namely AML1-ETO9a, c-Myc and MLL-AF9, were able to induce expression of certain snoRNAs. In contrast, global reduction of C/D box snoRNAs and subsequent reduced 2′-O-Me of rRNA ultimately affected secondary transplantations in mice, a clear sign of impaired self-renewal capacity of AML cells. Moreover, the set of snoRNAs SNORD14D, SNORD14E, SNORD20, SNORD32A, SNORD34, SNORD35A, SNORD43, SNORD53, SNORD74A and SNORD104 was identified to be strongly expressed in primary AML1-ETO+ samples with high LSC content. This snoRNA signature was also associated with poorer response to initial chemotherapy treatment [48].

### 2.2. Acute Leukemia: Focus on Acute Lymphoblastic Leukemia (ALL)

In analogy to AML, defects in essential components of the translation machinery have been reported in acute lymphoblastic leukemia (ALL). In T-cell acute lymphoblastic leukemia (T-ALL), several studies have revealed mutations in ribosomal proteins, vital components for correct ribosome function, which hindered translational fidelity and normal ribosome biogenesis [63,64,65]. T-ALL is related to leukemic transformation of lymphoid progenitors and deletion of the long arm of chromosome 6 (del6q) is a frequent chromosomal aberration found in T-ALL as well as in lymphoblastic lymphoma patients [66,67,68]. To elucidate the underlying molecular effectors of this chromosome rearrangement that accelerates T-ALL progression *in vivo*, a study identified affected genes located at the deleted genomic region. One of these regions was the small nucleolar host gene 5 (*SNHG5*) which also encodes two C/D box snoRNAs within its introns, SNORD50A and SNORD50B (also known as U50A and U50B) [69]. These two snoRNAs were previously reported to regulate ribosomal biogenesis through site-specific 2′-O-Me of pre-rRNAs [3]. Gachet and colleagues described these two snoRNAs acting as tumour suppressor genes reducing ribosomal functions and that the deletion induced leukemic transformation of LSCs in late stages of T-ALL. They hypothesised that this genomic alteration influenced the translation of key mitochondrial factors, resulted in reduced oxidative phosphorylation and aberrantly affected self-renewal capacity of normal HSCs. The genomic location of SNORD50 already pointed to its potential involvement in malignancies, since the snoRNA gene is affected by the recurrent breakpoint t(3;6) (q27;q15) involved in human B-cell lymphoma [70]. In accordance, the physiological importance of mouse SNORD50 (mU50) and its possible link in promoting tumourigenesis was also investigated [71]. By specific genomic deletion of mU50, the authors observed an increase in abnormal events in the lymphoid organs, including differentially regulated tissue-specific heat shock proteins.

As seen in AML, high expression of SNORD116 in patients with childhood B-cell precursor lymphoblastic leukemia (BCP-ALL) carrying ERG-related genetic aberrations was reported [72]. BCP-ALL is the most common subtype of ALL and importantly, patients with ERG-related alterations show a good response to standard therapies. The specific upregulation of a set of snoRNAs (SNORD64, SNORD107, and SNORD109A together with the SNORD116 cluster) was reported and was able to discriminate between ERG-related and non-ERG-related in BCP-ALL samples. Interestingly, these differentially regulated snoRNAs map to the 15q11.2 genomic region involved in the Prader–Willi Syndrome (PWS), a thoroughly studied locus that transcribes several orphan snoRNAs [73,74]. The dysregulation of components of the rRNA methylation complex was also observed in pediatric BCP-ALL. Here, snoRNA-associated FBL and NOP56 proteins were upregulated by modulation of the upstream effector c-Myc. Myc has been previously reported to positively affect levels of C/D box snoRNAs; as for p53, a negative impact on snoRNA function has been reported [48,75,76]. Additionally, SNORD35B and SNORD46 showed elevated levels in BCL-ALL patients with relapse compared to patients that maintained complete remission [77].

### 2.3. Chronic Lymphocytic Leukemia (CLL)

Although most of the patients with chronic lymphocytic leukemia (CLL) survive for many years even without treatment, others present an aggressively and rapidly evolving disease. This highlights the need for biomarkers that help to distinguish patients that can potentially develop more acute stages of CLL from those with chronic and stable stages [78]. This prompted scientists to identify novel biomarkers that predict the clinical outcome of CLL patients in early stages of the disease [79]. In this report, expression profiles of sno/scaRNAs in CLL cells were compared to their normal B-cell counterparts from patients with different karyotypes. SNORA6, SNORA31, SNORA62 and SNORA71C showed downregulation in CLL cells irrespective of the molecular subgroup of the patient. Interestingly, SNORA31 downregulation correlated with reduced expression of its host gene *TPT1*, a protein known to regulate stemness by influencing TP53-tumour-suppressor function [80]. SNORA70F was also reported in this study to be downregulated in CLL patients with adverse prognostic markers such as cytogenetically normal IGHV status, del11, ZAP70 or CD38+ [79]. The downregulation of this snoRNA also correlated with reduced expression of its host gene *COBLL1*, a gene whose downregulation is associated with poor prognosis in CLL patients [81]. Ronchetti et al. proposed an independent 2-snoRNA signature for predicting different prognostic groups in Binet stage-A CLL patients. High-risk CLL patients showed elevated expression levels of at least one of the two snoRNAs SNORA74A and SNORD116-18 [79]. So far, little is known about the exact function of SNORA74A although studies have connected its biological role to multiple myeloma [82]. SNORD116-18 is transcribed in a cluster of several copies located at the *SNURF-SNRPN* locus, previously mentioned to host several orphan snoRNAs.

Moreover, another study in CLL investigated snoRNA expression profiles in CLL patients with different IGHV mutational status and common mutations in TP53, NOTCH1 and SF3B1 [83]. In contrast with Ronchetti et al. [79], they could not identify specific snoRNA signatures associated with mutations that impact the outcome of CLL patients even though unsupervised analyses revealed a snoRNA profile (SNORD35B, SNORD71, SNORD116-11 and SNORD116-25) that could robustly discriminate between healthy B-cells and leukemic cells in CLL samples. They also identified a set of 20 snoRNAs overexpressed among IGHV-mutated CLL patients with shorter treatment-free survival rates, a widely used parameter to monitor disease progression in CLL. This signature included SNORA12, SNORA22, SNORA27, SNORA56, SNORA64, SNORA69, SNORA70, SNORA74A, SNORA80, SNORA84, SNORD1A, SNORD1B, SNORD8, SNOR18, SNORD30, SNORD32A, SNORD34, SNORD105B, SNORD110 and SCARNA8. However, only seven of these snoRNAs were correlated with increased proliferation in CLL primary cells *in vitro* when compared to normal B-cells and two of them (SNORA80 and SNORD1A) were downregulated upon induced proliferation. This suggests that the proposed snoRNA signature does not only reflect the potential of CLL proliferation but also the associated potential for leukemogenesis in IGHV-mutated patients.

### 2.4. Peripheral T-Cell Lymphoma (PTCL)

Revolutionary advances in the treatment and prognosis of human lymphomas have occurred despite unquestionably favouring patients with B-cell lymphomas. Peripheral T-cell lymphoma (PTCL) is a complex and heterogeneous type of non-Hodgkin lymphoma whose classification is divided in PTCL-not otherwise specified (PTCL-NOS), angio-immunoblastic T-cell lymphoma (AITL) and anaplastic large cell lymphoma (ALCL) with or without anaplastic lymphoma kinase genetic abnormalities (ALK+ ALCL and ALK− ALCL) and other less common subtypes. The current diagnostic issue is to identify patients with adverse and very adverse prognosis given that only 20% of patients are eligible for standard chemotherapy treatment [84,85]. Although molecular signatures, including coding genes, have been developed in order to refine the different PTCL subtypes [86], others are not clearly distinguishable, such as between PTCL-NOS and ALK− ALCL. For this reason, a study investigated if snoRNA-expression profiles were relevant for PTCL diagnosis and prognostication [87]. Supervised analyses revealed that using snoRNA U3 as a single marker was sufficient to discriminate between ALK+ and ALK− ALCL tumours. This snoRNA is produced as individual transcription unit and is connected to the p53 pathway [88,89]. Although U3 includes C/D box-like motifs, it is not involved in 2′-O-Methylation of rRNA but rather is directing site-specific cleavage of rRNAs [90]. Moreover, the studies identified a robust snoRNA signature for both AITL and PTCL-NOS capable of classifying these patients depending on their outcome. The signature included overexpressed snoRNAs (SNORA12, SNORD117, HBII-142, HBII-239, ACA54, U90 and U55) associated with prolonged overall survival. HBII-239 was suggested to be the strongest biomarker and its overexpression had also a negative effect on T-cell lymphoma growth [87].

### 2.5. Multiple Myeloma (MM)

Multiple myeloma is a fatal hematological malignancy that affects B-lineage plasma cells. Chromosomal aberrations are frequently observed among patients with MM and the t(4;14) (p16.3;q32.3) translocation is commonly associated with shortened overall survival [91,92]. This chromosomal rearrangement consists of the transposition of the immunoglobulin heavy chain region enhancer elements to the 5′ region of the nuclear receptor binding SET domain protein 2 gene (*NSD2*, also known as *WHSC1*) leading to ectopic overexpression in MM cells [93]. In order to improve the understanding of how the t(4;14) alteration contributes to myelomagenesis, a novel H/ACA box snoRNA called ACA11 (also known as SCARNA22) encoded within the introns of the *WHSC1* gene was identified [94]. ACA11 was demonstrated to be robustly overexpressed among t(4;14)-positive MM cell lines/patients as well as in subgroups of patients with bladder, colon and esophageal cancer. The function of this snoRNA was associated with proteins implicated in RNA processing as *in vitro* studies showed association with RNA splicing factors (SF3B1, SF3B2 and SFPQ), RNA helicase (DHX9), RNA-specific deaminase (ADAR) and proteins of the heterogeneous nuclear ribonucleoprotein family (HNRNP). ACA11 was also found to mediate downregulation of certain ribosomal protein transcripts, such as RPL13A, even though exogenous overexpression of ACA11 did not affect the formation of the ribosome machinery. Moreover, ACA11 overexpression in t(4;14)-negative MM cells reduced oxidative stress, increased proliferation and chemoresistance. Together, this study identified ACA11 snoRNA as a key component in translocation-associated MM pathogenesis and a potential therapeutic target for t(4;14)-positive MM patients. Later, the function of ACA11 in MM cells was investigated in more detail [95]. This study demonstrated for the first time that ACA11 upregulates ribosome biogenesis in a ROS-dependent manner. An increased number and size of nucleoli in human t(4;14)-positive MM samples and ACA11-overexpressing MM cells was observed. Furthermore, it was hypothesised that ACA11 increases the available cytosolic ribosomes with subsequent increase in protein synthesis rate to support cell growth. Interestingly, ACA11 overexpression also led to a notable sensitivity to the proteasome inhibitor Btz, a commonly used therapy for MM patients that has improved the survival rate of MM patients at all stages [96].

In order to elucidate the pathogenic role of certain snoRNAs in MM, sno/scaRNA expression profiles were investigated in different cohorts of MM and secondary plasma cell leukemia (sPCL) patients compared with non-neoplastic counterparts [82]. In agreement with previous studies that showed global downregulation of snoRNAs in acute myeloid and lymphoblastic leukemias, downregulation of SNORD32A and SNORA42 was also reported in sPCL [51]. It was hypothesised that SNORD32A downregulation may induce resistance to endoplasmic reticulum stress-induced response as it was demonstrated *in vitro* in mouse models [97]. In the case of SNORD42, correlations were only found that pointed towards (1) the region where this snoRNA is located (chr1q22), commonly amplified in plasma cell dyscrasias and (2) that SNORA42 activation had an oncogenic role in lung tumourigenesis in non-small cell lung cancer cell lines [98]. A specific upregulation of a snoRNA signature in MM patients with hyperploid karyotype was also reported, commonly associated with trisomies and showing favourable clinical outcome [99]. This molecular group is characterised also by a global upregulation of the translational machinery including components of the ribosome biogenesis pathway and proteins regulating processing and modification of rRNAs [100]. In this regard, SNORD36C, SNORD63, SNORD95 and SNORA40 were shown to be downregulated in this molecular subgroup of MM patients in correlation with reduced expression of their host genes *RPL7A*, *HSPA9B*, *TACK1* and *TAF1D*, respectively [101,102]. Furthermore, the study reported another signature consisting of 14 snoRNAs (SNORA74A, SNORD101, SNORD115-24, 25, 31, 32, 7, SNORD116-22, 23, 25, 29, SNORD24, SNORD36C, SNORD8, SCARNA22) specific for the TC2 subgroup of MM patients [82]. The TC2 group is characterised by the overexpression of genes involved in protein biosynthesis [103]. In this study, SNORD115 and SNORD116 variants were correlated with DNA copy number of the MM patients [82]. Interestingly, a shorter processed form of orphan SNORD115 was found to regulate alternative splicing of the serotonin receptor mRNA HTR2C [104].

## 3. Conclusions and Future Perspective

**Figure 2:**
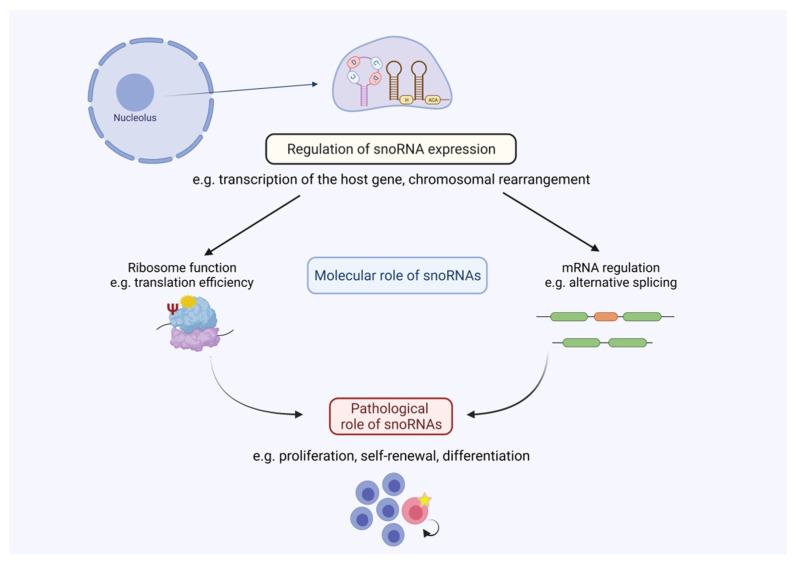
SnoRNA-mediated regulation in hematological malignancies. Altered snoRNA expression leads to changes in snoRNA abundance, which influences cellular processes such as ribosome function and/or mRNA expression. These molecular processes can control pathological processes as seen in, for example, the regulation of proliferation or self-renewal capacity in hematological diseases. Figure created with BioRender.com (accessed on 20 October 2021).

It has become increasingly evident that snoRNAs show a strong connection to hematological diseases (summarised in Table 1). Especially due to analysis of global ncRNA expression patterns, snoRNAs were found to be deregulated in a variety of these clinical contexts. Accordingly, expression of snoRNAs seems to be regulated at different levels. Hence, snoRNA expression can be indirectly influenced by enhanced transcription of their host genes facilitated by oncogenic transcription factors, such as c-Myc in AML (see Figure 2). On the other hand, snoRNA expression can directly be influenced by tumour-specific genomic alterations as it is evident for snoRNA downregulation in, for example, B-cell lymphoma, by destruction of the U50 locus or chromosomal gains e.g., SNORD42 in multiple myeloma. However, in a variety of clinical contexts, the underlying mechanism causing snoRNA deregulation is not well understood. Future studies should emphasise identifying molecular pathways, transcription programs and genomic setups that lead to altered snoRNA expression in hematological diseases.

Regarding snoRNA function, most of the studies focus on prognostic parameters and expression patterns. Sadly, little is known about fundamental cellular mechanisms guiding snoRNA-mediated control of gene expression (see Figure 2). It can be assumed that most of the snoRNA-directed cellular functions involve scaffolding roles that include association with RNA-binding proteins, as it was described for other small ncRNAs [105]. Some studies could correlate changed snoRNA abundance with the rRNA modification status, generally being the most prevalent mechanism associated with snoRNA function (e.g., [48]). In cases where orphan snoRNAs are deregulated in the clinical context, it can be hypothesised which physiological targets might trigger cellular responses. For SNORD116, a function in alternative splicing was proposed, which clearly differs from the canonical role of snoRNAs. It remains to be elucidated on a global scale which molecular targets snoRNAs might have especially in the hema-oncological context. Genome wide experimental procedures, such as RiboMeth sequencing and CLASH, could provide novel insights into the global snoRNA-mediated control of gene expression. Specific targets for either rRNAs or mRNAs could then be further validated and shed light on the plethora of snoRNA targets especially in hematological diseases.

The investigation of snoRNA biology in the context of diseases is still a new topic, where the majority of manuscripts have been published within the last ten years, although snoRNAs themselves have been known for decades. Nevertheless, we still do not fully understand how snoRNAs contribute to disease progression in the hematological context (see Figure 2). Over a long period of time, these analyses were hampered by an insufficient number of methods to directly modulate snoRNA expression. Novel technologies have now been established (ASOs/GapmeRs and CRISPR/Cas9) that also allow the specific modulation of snoRNA expression. We expect that these technologies will be used more frequently in the future to investigate the cellular phenotypes (e.g., proliferation and self-renewal capacity) associated with snoRNA depletion. Additionally, ASOs for instance, can be applied *in vivo* [36]. Hence, this opens the possibility to analyse the targeting of oncogenic snoRNAs as a novel therapeutic approach especially in hematological diseases. Like for other ncRNAs, conservation of snoRNAs across species is not always consistent. This makes the investigation of snoRNA function *in vivo*, e.g., by the use of genetic mouse models, much harder. There are cases where snoRNAs are conserved among species and functions in mice could be connected to phenotypes seen in humans (e.g., U50 [71]). In cases where snoRNAs are not conserved, xenotransplantation of modified cells (e.g., with CRISPR/Cas9) into appropriate mouse strains could be used to determine the *in vivo* and clinical role of snoRNAs. We envision that future studies will provide more evidence about the multifaceted role of snoRNAs beyond hematological diseases, since researchers have just begun to shed light on this powerful ncRNA regulator connected to disease development and progression.

## Figures and Tables

**Table 1:** Summary of snoRNAs with pathological relevance in hematological diseases. ND—not determined.

snoRNA	Type	Genomic Location	Expression	Putative Targets/Pathways	Relevance in Disease Context	Reference
**SNORD112,113,114**	C/D	14q32	Overexpression	ND	Acute Promyelocytic Leukemia	[51]
**SNORD114-1**	C/D	14q32	Overexpression	Inhibition of cell cycle via Rb pathway	Acute Promyelocytic Leukemia	[51]
**SNORD113-3,-4; SNORD114-2,-3**	C/D	14q32	Overexpression	ND	Acute Promyelocytic Leukemia	[51]
**SNORD115, SNORD116**	C/D	14q32	Overexpression	SNORD115 shorter isoform regulates the alternative splicing of the 5HT-2C serotonin receptor pre-mRNA	Higher expression in CD34^+^ progenitor cells	[27,104]
**SCARNA15**	scaRNA	15q25	Downregulation	Ψ in U2 spliceosomal RNA	Decreased in AML samples compared to normal CD34^+^ cells	[51]
**SNORA21**	H/ACA	17q12	Downregulation	Modification of PTC of ribosome	Decreased in AML samples compared to normal CD34^+^ cells	[27]
**SNORA36**	H/ACA	Xq28	Downregulation	Modification of ISB of ribosome	Decreased in AML samples compared to normal CD34^+^ cells	[27]
**SNORD42A**	C/D	17q11	Overexpression	Bound to NPM1. 2′-O-Me of 18S rRNA	Highly expressed in primary AML blasts. SNORD42 downregulation in sPCL patients.	[59,82]
**SNORD15** **SNORD47** **SNORD52** **SNORD58** **SNORD104**	C/D	11q13 1q25 6p21 14q21 17q23	Enriched in AML samples with NPMc+ mutations	Bound to NPM1 2′-O-Me of rRNAs	Regulation cellular growth, differentiation and HSCs maintenance. SNORD104 highly expressed in AML1-ETO+ samples with high LSC content.	[48,61]
**SNORD14D** **SNORD35A**	C/D	11q23 19q13	AML1-ETO induced expression	2′-O-Me of 18S and 28S rRNA	Knockout reduced colony formation in AML cells and delayed leukemogenesis *in vivo* High expression in AML1-ETO+ samples with high LSC content	[48]
**SNORD34** **SNORD43**	C/D	19q13 22q13	AML1-ETO induced expression	2′-O-Me of 18S and 28S rRNA	Reduced expression impairs clonogenic growth of AML cells. High expression in AML1-ETO+ samples with high LSC content	[48]
**SNORD14E** **SNORD20** **SNORD32A** **SNORD53** **SNORD74**	C/D	11q23 2q37 19q13 2p23 1q25	AML1-ETO induced expression	-	High expression in AML1-ETO+ samples with high LSC content. SNORD32A downregulated in sPCL patients	[48]
**SNORD50A, B**	C/D	6q14	Deletion	2′-O-Me of 28S pre- rRNAs	Haploinsufficiency plays a role in late-stage T-cell leukemia and B-cell lymphoma	[69,70]
**SNORD116-11, -14, -15, -16, -17, -18, -20, -21, -22, -23, -24, -27,** **SNORD64** **SNORD107** **SNORD109A**	C/D	15q11	Overexpression	ND	Distinguish BCP-ALL patients with and without ERG intragenic deletions	[72]
**SNORD35B** **SNORD46**	C/D	19q13 1p34	Overexpression	ND	Identification of BCP-ALL patients at early diagnosis who fail to therapy. SNORD35B is differentially regulated in CLL cells.	[77,83]
**SNORA6** **SNORA31** **SNORA62** **SNORA70F** **SNORA71C**	H/ACA	3p22 13q14 3p22 2q24 20q11	Downregulation	ND	Lower expression in CLL cells compared to their B-cell counterparts	[79]
**SNORA74A** **SNORD116-18**	H/ACA C/D	5q31 15q11	Overexpression	ND	Identification of high-risk CLL patients. SNORA74A included in a signature for identifying a subgroup of MM patients	[79,82]
**SNORD71** **SNORD116-11, -25**	C/D	16q22 15q11	Overexpression Downregulation	ND	Discriminates between normal B-cells and CLL cells. SNORD71 overexpression is correlated with prolonged OS in subgroups of PTCL patients	[83,87]
**SNORD1A** **SNORA80**	C/D H/ACA	17q25 21q22		ND	In IGHV-mutated CLL patients, correlates with shorter treatment-free survival	[83]
**snoRNA U3**	C/D	8p21	Overexpression	Interaction with p53 pathway	Upregulated in ALK+ PTCL tumours	[87]
**SNORA12** **SNORD117** **HBII-142/SNORD66** **ACA54/SNORA54** **snoRNA U55/SNORD55** **snoRNA U90/SCARNA7**	H/ACA C/D C/D H/ACA C/D scaRNA	10q24 6p21 3q27 11p15 1p34 3q25	Overexpression	ND	Overexpression of the signature correlates with prolonged OS in non-ALCL PTCL patients	[87]
**ACA11/SCARNA22**	scaRNA	4p16	Overexpression in t(4;14)-positive MM patients	RNA processing and downregulation of RPL13A	Linked to pathogenesis in MM through regulation of oxidative stress and chemosensitivity	[94,95]
**SNORD36C** **SNORD63** **SNORD95** **SNORA40**	C/D C/D C/D H/ACA	9q34 5q31 5q35 11q21	Downregulation		Downregulation correlates with molecular signature in a subgroup of MM patients	[82]
**SNORD24** **SNORD36** **SNORD101** **SNORD115-7,-24,-25,-31,-32** **SNORD116-22,-23, -25, -29,**	C/D C/D C/D C/D C/D	9q34 9q34 6q23 15q11 15q11	Overexpression	SNORD115 shorter isoform regulates the alternative splicing of the 5HT-2C serotonin receptor pre-mRNA	Signature for identification in a subgroup of MM patients (TC2)	[82,104]

## References

[1] Deveson I.W., Hardwick S.A., Mercer T.R., Mattick J.S. (2017). The Dimensions, Dynamics, and Relevance of the Mammalian Noncoding Transcriptome. Trends Genet..

[2] Reddy R., Busch H. (1988). Small Nuclear RNAs: RNA Sequences, Structure, and Modifications.

[3] Kiss-Laszlo Z., Henry Y., Bachellerie J.P., Caizergues-Ferrer M., Kiss T. (1996). Site-specific ribose methylation of preribosomal RNA: A novel function for small nucleolar RNAs. Cell.

[4] Tollervey D., Kiss T. (1997). Function and synthesis of small nucleolar RNAs. Curr. Opin. Cell Biol..

[5] Weinstein L.B., Steitz J.A. (1999). Guided tours: From precursor snoRNA to functional snoRNP. Curr. Opin. Cell Biol..

[6] Esteller M. (2011). Non-coding RNAs in human disease. Nat. Rev. Genet..

[7] Kiss T. (2002). Small nucleolar RNAs: An abundant group of noncoding RNAs with diverse cellular functions. Cell.

[8] Liang J., Wen J., Huang Z., Chen X.P., Zhang B.X., Chu L. (2019). Small Nucleolar RNAs: Insight Into Their Function in Cancer. Front. Oncol.

[9] Decatur W.A., Fournier M.J. (2002). rRNA modifications and ribosome function. Trends Biochem. Sci..

[10] Sharma S., Lafontaine D.L.J. (2015). ‘View From A Bridge’: A New Perspective on Eukaryotic rRNA Base Modification. Trends Biochem. Sci..

[11] Jorjani H., Kehr S., Jedlinski D.J., Gumienny R., Hertel J., Stadler P.F., Zavolan M., Gruber A.R. (2016). An updated human snoRNAome. Nucleic Acids Res..

[12] Massenet S., Bertrand E., Verheggen C. (2017). Assembly and trafficking of box C/D and H/ACA snoRNPs. RNA Biol..

[13] Monaco P.L., Marcel V., Diaz J.J., Catez F. (2018). 2′-O-Methylation of Ribosomal RNA: Towards an Epitranscriptomic Control of Translation?. Biomolecules.

[14] Dieci G., Preti M., Montanini B. (2009). Eukaryotic snoRNAs: A paradigm for gene expression flexibility. Genomics.

[15] Kufel J., Grzechnik P. (2019). Small Nucleolar RNAs Tell a Different Tale. Trends Genet..

[16] Davis D.R. (1995). Stabilization of RNA stacking by pseudouridine. Nucleic Acids Res..

[17] King T.H., Liu B., McCully R.R., Fournier M.J. (2003). Ribosome structure and activity are altered in cells lacking snoRNPs that form pseudouridines in the peptidyl transferase center. Mol. Cell.

[18] Liang X.H., Liu Q., Fournier M.J. (2007). rRNA modifications in an intersubunit bridge of the ribosome strongly affect both ribosome biogenesis and activity. Mol. Cell.

[19] Piekna-Przybylska D., Przybylski P., Baudin-Baillieu A., Rousset J.P., Fournier M.J. (2008). Ribosome performance is enhanced by a rich cluster of pseudouridines in the A-site finger region of the large subunit. J. Biol. Chem..

[20] Tycowski K.T., Shu M.D., Kukoyi A., Steitz J.A. (2009). A conserved WD40 protein binds the Cajal body localization signal of scaRNP particles. Mol. Cell.

[21] Deryusheva S., Choleza M., Barbarossa A., Gall J.G., Bordonne R. (2012). Post-transcriptional modification of spliceosomal RNAs is normal in SMN-deficient cells. RNA.

[22] Reichow S.L., Hamma T., Ferre-D’Amare A.R., Varani G. (2007). The structure and function of small nucleolar ribonucleoproteins. Nucleic Acids Res..

[23] Matera A.G., Terns R.M., Terns M.P. (2007). Non-coding RNAs: Lessons from the small nuclear and small nucleolar RNAs. Nat. Rev. Mol. Cell Biol..

[24] Williams G.T., Farzaneh F. (2012). Are snoRNAs and snoRNA host genes new players in cancer?. Nat. Rev. Cancer.

[25] Lykke-Andersen S., Chen Y., Ardal B.R., Lilje B., Waage J., Sandelin A., Jensen T.H. (2014). Human nonsense-mediated RNA decay initiates widely by endonucleolysis and targets snoRNA host genes. Genes Dev..

[26] Bratkovic T., Bozic J., Rogelj B. (2020). Functional diversity of small nucleolar RNAs. Nucleic Acids Res..

[27] Warner W.A., Spencer D.H., Trissal M., White B.S., Helton N., Ley T.J., Link D.C. (2018). Expression profiling of snoRNAs in normal hematopoiesis and AML. Blood Adv..

[28] Zorn P., Misiak D., Gekle M., Kohn M. (2021). Identification and initial characterization of POLIII-driven transcripts by msRNA-sequencing. RNA Biol..

[29] Bazeley P.S., Shepelev V., Talebizadeh Z., Butler M.G., Fedorova L., Filatov V., Fedorov A. (2008). snoTARGET shows that human orphan snoRNA targets locate close to alternative splice junctions. Gene.

[30] Tafer H., Kehr S., Hertel J., Hofacker I.L., Stadler P.F. (2010). RNAsnoop: Efficient target prediction for H/ACA snoRNAs. Bioinformatics.

[31] Kehr S., Bartschat S., Stadler P.F., Tafer H. (2011). PLEXY: Efficient target prediction for box C/D snoRNAs. Bioinformatics.

[32] Chen C.L., Perasso R., Qu L.H., Amar L. (2007). Exploration of pairing constraints identifies a 9 base-pair core within box C/D snoRNA-rRNA duplexes. J. Mol. Biol..

[33] Gumienny R., Jedlinski D.J., Schmidt A., Gypas F., Martin G., Vina-Vilaseca A., Zavolan M. (2017). High-throughput identification of C/D box snoRNA targets with CLIP and RiboMeth-seq. Nucleic Acids Res..

[34] Kudla G., Granneman S., Hahn D., Beggs J.D., Tollervey D. (2011). Cross-linking, ligation, and sequencing of hybrids reveals RNA-RNA interactions in yeast. Proc. Natl. Acad. Sci. USA.

[35] Sledziowska M., Jones M., Maghrabi R.A., Hebenstreit D., Garcia P., Grzechnik P. Non-coding RNAs Associated with Prader-Willi Syndrome Regulate Transcription of Neurodevelopmental Genes in Human Induced Pluripotent Stem Cells. bioRxiv.

[36] Liang X.H., Vickers T.A., Guo S., Crooke S.T. (2011). Efficient and specific knockdown of small non-coding RNAs in mammalian cells and in mice. Nucleic Acids Res..

[37] Filippova J.A., Matveeva A.M., Zhuravlev E.S., Balakhonova E.A., Prokhorova D.V., Malanin S.J., Shah Mahmud R., Grigoryeva T.V., Anufrieva K.S., Semenov D.V. (2019). Are Small Nucleolar RNAs “CRISPRable”? A Report on Box C/D Small Nucleolar RNA Editing in Human Cells. Front. Pharmacol..

[38] Jagannathan-Bogdan M., Zon L.I. (2013). Hematopoiesis. Development.

[39] Cheshier S.H., Morrison S.J., Liao X., Weissman I.L. (1999). In vivo proliferation and cell cycle kinetics of long-term self-renewing hematopoietic stem cells. Proc. Natl. Acad. Sci. USA.

[40] Passegue E., Wagers A.J., Giuriato S., Anderson W.C., Weissman I.L. (2005). Global analysis of proliferation and cell cycle gene expression in the regulation of hematopoietic stem and progenitor cell fates. J. Exp. Med..

[41] Pinho S., Frenette P.S. (2019). Haematopoietic stem cell activity and interactions with the niche. Nat. Rev. Mol. Cell Biol..

[42] Signer R.A., Magee J.A., Salic A., Morrison S.J. (2014). Haematopoietic stem cells require a highly regulated protein synthesis rate. Nature.

[43] Spevak C.C., Elias H.K., Kannan L., Ali M.A.E., Martin G.H., Selvaraj S., Eng W.S., Ernlund A., Rajasekhar V.K., Woolthuis C.M. (2020). Hematopoietic Stem and Progenitor Cells Exhibit Stage-Specific Translational Programs via mTOR- and CDK1-Dependent Mechanisms. Cell Stem Cell.

[44] Fazi F., Fatica A. (2021). Regulation of Ribosome Function by RNA Modifications in Hematopoietic Development and Leukemia: It Is Not Only a Matter of m(6)A. Int. J. Mol. Sci..

[45] Hidalgo San Jose L., Sunshine M.J., Dillingham C.H., Chua B.A., Kruta M., Hong Y., Hatters D.M., Signer R.A.J. (2020). Modest Declines in Proteome Quality Impair Hematopoietic Stem Cell Self-Renewal. Cell Rep..

[46] Herter E.K., Stauch M., Gallant M., Wolf E., Raabe T., Gallant P. (2015). snoRNAs are a novel class of biologically relevant Myc targets. BMC Biol..

[47] Cai X., Gao L., Teng L., Ge J., Oo Z.M., Kumar A.R., Gilliland D.G., Mason P.J., Tan K., Speck N.A. (2015). Runx1 Deficiency Decreases Ribosome Biogenesis and Confers Stress Resistance to Hematopoietic Stem and Progenitor Cells. Cell Stem Cell.

[48] Zhou F., Liu Y., Rohde C., Pauli C., Gerloff D., Kohn M., Misiak D., Baumer N., Cui C., Gollner S. (2017). AML1-ETO requires enhanced C/D box snoRNA/RNP formation to induce self-renewal and leukaemia. Nat. Cell Biol..

[49] Terwilliger T., Abdul-Hay M. (2017). Acute lymphoblastic leukemia: A comprehensive review and 2017 update. Blood Cancer J..

[50] Dohner H., Weisdorf D.J., Bloomfield C.D. (2015). Acute Myeloid Leukemia. N. Engl. J. Med..

[51] Valleron W., Laprevotte E., Gautier E.F., Quelen C., Demur C., Delabesse E., Agirre X., Prosper F., Kiss T., Brousset P. (2012). Specific small nucleolar RNA expression profiles in acute leukemia. Leukemia.

[52] Martens J.H., Brinkman A.B., Simmer F., Francoijs K.J., Nebbioso A., Ferrara F., Altucci L., Stunnenberg H.G. (2010). PML-RARalpha/RXR Alters the Epigenetic Landscape in Acute Promyelocytic Leukemia. Cancer Cell.

[53] Alvarez-Dominguez J.R., Hu W., Gromatzky A.A., Lodish H.F. (2014). Long noncoding RNAs during normal and malignant hematopoiesis. Int. J. Hematol..

[54] Dostalova Merkerova M., Krejcik Z., Votavova H., Belickova M., Vasikova A., Cermak J. (2011). Distinctive microRNA expression profiles in CD34+ bone marrow cells from patients with myelodysplastic syndrome. Eur. J. Hum. Genet..

[55] Cohen Y., Hertzog K., Reish O., Mashevich M., Garach-Jehoshua O., Bar-Chaim A., Trakhtenbrot L., Kornberg A. (2012). The increased expression of 14q32 small nucleolar RNA transcripts in promyelocytic leukemia cells is not dependent on PML-RARA fusion gene. Blood Cancer J..

[56] Liuksiala T., Teittinen K.J., Granberg K., Heinaniemi M., Annala M., Maki M., Nykter M., Lohi O. (2014). Overexpression of SNORD114-3 marks acute promyelocytic leukemia. Leukemia.

[57] Donmez G., Hartmuth K., Luhrmann R. (2004). Modified nucleotides at the 5’ end of human U2 snRNA are required for spliceosomal E-complex formation. RNA.

[58] Warner W.A. (2019). Expression and Function of snoRNAs in Acute Myeloid Leukemia. Arts Sci. Electron. Theses Diss..

[59] Pauli C., Liu Y., Rohde C., Cui C., Fijalkowska D., Gerloff D., Walter C., Krijgsveld J., Dugas M., Edemir B. (2020). Site-specific methylation of 18S ribosomal RNA by SNORD42A is required for acute myeloid leukemia cell proliferation. Blood.

[60] Borer R.A., Lehner C.F., Eppenberger H.M., Nigg E.A. (1989). Major nucleolar proteins shuttle between nucleus and cytoplasm. Cell.

[61] Nachmani D., Bothmer A.H., Grisendi S., Mele A., Bothmer D., Lee J.D., Monteleone E., Cheng K., Zhang Y., Bester A.C. (2019). Germline NPM1 mutations lead to altered rRNA 2’-O-methylation and cause dyskeratosis congenita. Nat. Genet..

[62] Falini B., Mecucci C., Tiacci E., Alcalay M., Rosati R., Pasqualucci L., La Starza R., Diverio D., Colombo E., Santucci A. (2005). Cytoplasmic nucleophosmin in acute myelogenous leukemia with a normal karyotype. N. Engl. J. Med..

[63] De Keersmaecker K., Atak Z.K., Li N., Vicente C., Patchett S., Girardi T., Gianfelici V., Geerdens E., Clappier E., Porcu M. (2013). Exome sequencing identifies mutation in CNOT3 and ribosomal genes RPL5 and RPL10 in T-cell acute lymphoblastic leukemia. Nat. Genet..

[64] Sulima S.O., Patchett S., Advani V.M., De Keersmaecker K., Johnson A.W., Dinman J.D. (2014). Bypass of the pre-60S ribosomal quality control as a pathway to oncogenesis. Proc. Natl. Acad. Sci. USA.

[65] Girardi T., De Keersmaecker K. (2015). T-ALL: ALL a matter of Translation?. Haematologica.

[66] Aifantis I., Raetz E., Buonamici S. (2008). Molecular pathogenesis of T-cell leukaemia and lymphoma. Nat. Rev. Immunol..

[67] Remke M., Pfister S., Kox C., Toedt G., Becker N., Benner A., Werft W., Breit S., Liu S., Engel F. (2009). High-resolution genomic profiling of childhood T-ALL reveals frequent copy-number alterations affecting the TGF-beta and PI3K-AKT pathways and deletions at 6q15-16.1 as a genomic marker for unfavorable early treatment response. Blood.

[68] Bonn B.R., Rohde M., Zimmermann M., Krieger D., Oschlies I., Niggli F., Wrobel G., Attarbaschi A., Escherich G., Klapper W. (2013). Incidence and prognostic relevance of genetic variations in T-cell lymphoblastic lymphoma in childhood and adolescence. Blood.

[69] Gachet S., El-Chaar T., Avran D., Genesca E., Catez F., Quentin S., Delord M., Therizols G., Briot D., Meunier G. (2018). Deletion 6q Drives T-cell Leukemia Progression by Ribosome Modulation. Cancer Discov..

[70] Tanaka R., Satoh H., Moriyama M., Satoh K., Morishita Y., Yoshida S., Watanabe T., Nakamura Y., Mori S. (2000). Intronic U50 small-nucleolar-RNA (snoRNA) host gene of no protein-coding potential is mapped at the chromosome breakpoint t(3;6)(q27;q15) of human B-cell lymphoma. Genes Cells.

[71] Soeno Y., Fujita K., Kudo T., Asagiri M., Kakuta S., Taya Y., Shimazu Y., Sato K., Tanaka-Fujita R., Kubo S. (2013). Generation of a mouse model with down-regulated U50 snoRNA (SNORD50) expression and its organ-specific phenotypic modulation. PLoS ONE.

[72] Vendramini E., Giordan M., Giarin E., Michielotto B., Fazio G., Cazzaniga G., Biondi A., Silvestri D., Valsecchi M.G., Muckenthaler M.U. (2017). High expression of miR-125b-2 and SNORD116 noncoding RNA clusters characterize ERG-related B cell precursor acute lymphoblastic leukemia. Oncotarget.

[73] Galiveti C.R., Raabe C.A., Konthur Z., Rozhdestvensky T.S. (2014). Differential regulation of non-protein coding RNAs from Prader-Willi Syndrome locus. Sci. Rep..

[74] Zahova S., Isles A.R. (2018). The Role of the Prader-Willi Syndrome Critical Interval for Epigenetic Regulation, Transcription and Phenotype. Epigenomes.

[75] Marcel V., Ghayad S.E., Belin S., Therizols G., Morel A.P., Solano-Gonzalez E., Vendrell J.A., Hacot S., Mertani H.C., Albaret M.A. (2013). p53 acts as a safeguard of translational control by regulating fibrillarin and rRNA methylation in cancer. Cancer Cell.

[76] Krastev D.B., Slabicki M., Paszkowski-Rogacz M., Hubner N.C., Junqueira M., Shevchenko A., Mann M., Neugebauer K.M., Buchholz F. (2011). A systematic RNAi synthetic interaction screen reveals a link between p53 and snoRNP assembly. Nat. Cell Biol..

[77] Ussowicz M., Marcel V., Long F.N.V., Kazanowska B., Diaz J.J., Wolowiec D. (2020). Analysis of the rRNA methylation complex components in pediatric B-cell precursor acute lymphoblastic leukemia: A pilot study. Adv. Clin. Exp. Med..

[78] Chiorazzi N., Rai K.R., Ferrarini M. (2005). Chronic lymphocytic leukemia. N. Engl. J. Med..

[79] Ronchetti D., Mosca L., Cutrona G., Tuana G., Gentile M., Fabris S., Agnelli L., Ciceri G., Matis S., Massucco C. (2013). Small nucleolar RNAs as new biomarkers in chronic lymphocytic leukemia. BMC Med. Genom..

[80] Amson R., Pece S., Marine J.C., Di Fiore P.P., Telerman A. (2013). TPT1/ TCTP-regulated pathways in phenotypic reprogramming. Trends Cell Biol..

[81] Mansouri L., Gunnarsson R., Sutton L.A., Ameur A., Hooper S.D., Mayrhofer M., Juliusson G., Isaksson A., Gyllensten U., Rosenquist R. (2012). Next generation RNA-sequencing in prognostic subsets of chronic lymphocytic leukemia. Am. J. Hematol..

[82] Ronchetti D., Todoerti K., Tuana G., Agnelli L., Mosca L., Lionetti M., Fabris S., Colapietro P., Miozzo M., Ferrarini M. (2012). The expression pattern of small nucleolar and small Cajal body-specific RNAs characterizes distinct molecular subtypes of multiple myeloma. Blood Cancer J..

[83] Berquet L., Valleron W., Grgurevic S., Quelen C., Zaki O., Quillet-Mary A., Davi F., Brousset P., Bousquet M., Ysebaert L. (2016). Small nucleolar RNA expression profiles refine the prognostic impact of IGHV mutational status on treatment-free survival in chronic lymphocytic leukaemia. Br. J. Haematol..

[84] Jaffe E.S. (2009). The 2008 WHO classification of lymphomas: Implications for clinical practice and translational research. Hematol. Am. Soc. Hematol. Educ. Program.

[85] Fiore D., Cappelli L.V., Broccoli A., Zinzani P.L., Chan W.C., Inghirami G. (2020). Peripheral T cell lymphomas: From the bench to the clinic. Nat. Rev. Cancer.

[86] Iqbal J., Weisenburger D.D., Greiner T.C., Vose J.M., McKeithan T., Kucuk C., Geng H., Deffenbacher K., Smith L., Dybkaer K. (2010). Molecular signatures to improve diagnosis in peripheral T-cell lymphoma and prognostication in angioimmunoblastic T-cell lymphoma. Blood.

[87] Valleron W., Ysebaert L., Berquet L., Fataccioli V., Quelen C., Martin A., Parrens M., Lamant L., de Leval L., Gisselbrecht C. (2012). Small nucleolar RNA expression profiling identifies potential prognostic markers in peripheral T-cell lymphoma. Blood.

[88] Dragon F., Gallagher J.E., Compagnone-Post P.A., Mitchell B.M., Porwancher K.A., Wehner K.A., Wormsley S., Settlage R.E., Shabanowitz J., Osheim Y. (2002). A large nucleolar U3 ribonucleoprotein required for 18S ribosomal RNA biogenesis. Nature.

[89] Hu L., Wang J., Liu Y., Zhang Y., Zhang L., Kong R., Zheng Z., Du X., Ke Y. (2011). A small ribosomal subunit (SSU) processome component, the human U3 protein 14A (hUTP14A) binds p53 and promotes p53 degradation. J. Biol. Chem..

[90] Clery A., Senty-Segault V., Leclerc F., Raue H.A., Branlant C. (2007). Analysis of sequence and structural features that identify the B/C motif of U3 small nucleolar RNA as the recognition site for the Snu13p-Rrp9p protein pair. Mol. Cell Biol..

[91] Fonseca R., Bergsagel P.L., Drach J., Shaughnessy J., Gutierrez N., Stewart A.K., Morgan G., Van Ness B., Chesi M., Minvielle S. (2009). International Myeloma Working Group molecular classification of multiple myeloma: Spotlight review. Leukemia.

[92] Munshi N.C., Anderson K.C., Bergsagel P.L., Shaughnessy J., Palumbo A., Durie B., Fonseca R., Stewart A.K., Harousseau J.L., Dimopoulos M. (2011). Consensus recommendations for risk stratification in multiple myeloma: Report of the International Myeloma Workshop Consensus Panel 2. Blood.

[93] Chesi M., Nardini E., Lim R.S., Smith K.D., Kuehl W.M., Bergsagel P.L. (1998). The t(4;14) translocation in myeloma dysregulates both FGFR3 and a novel gene, MMSET, resulting in IgH/MMSET hybrid transcripts. Blood.

[94] Chu L., Su M.Y., Maggi L.B., Lu L., Mullins C., Crosby S., Huang G., Chng W.J., Vij R., Tomasson M.H. (2012). Multiple myeloma-associated chromosomal translocation activates orphan snoRNA ACA11 to suppress oxidative stress. J. Clin. Investig..

[95] Oliveira V., Mahajan N., Bates M.L., Tripathi C., Kim K.Q., Zaher H.S., Maggi L.B., Tomasson M.H. (2019). The snoRNA target of t(4;14) in multiple myeloma regulates ribosome biogenesis. FASEB Bioadv..

[96] Dou Q.P., Zonder J.A. (2014). Overview of proteasome inhibitor-based anti-cancer therapies: Perspective on bortezomib and second generation proteasome inhibitors versus future generation inhibitors of ubiquitin-proteasome system. Curr. Cancer Drug Targets.

[97] Michel C.I., Holley C.L., Scruggs B.S., Sidhu R., Brookheart R.T., Listenberger L.L., Behlke M.A., Ory D.S., Schaffer J.E. (2011). Small nucleolar RNAs U32a, U33, and U35a are critical mediators of metabolic stress. Cell Metab..

[98] Mei Y.P., Liao J.P., Shen J., Yu L., Liu B.L., Liu L., Li R.Y., Ji L., Dorsey S.G., Jiang Z.R. (2012). Small nucleolar RNA 42 acts as an oncogene in lung tumorigenesis. Oncogene.

[99] Barila G., Bonaldi L., Grassi A., Martines A., Lico A., Macri N., Nalio S., Pavan L., Berno T., Branca A. (2020). Identification of the true hyperdiploid multiple myeloma subset by combining conventional karyotyping and FISH analysis. Blood Cancer J..

[100] Agnelli L., Fabris S., Bicciato S., Basso D., Baldini L., Morabito F., Verdelli D., Todoerti K., Lambertenghi-Deliliers G., Lombardi L. (2007). Upregulation of translational machinery and distinct genetic subgroups characterise hyperdiploidy in multiple myeloma. Br. J. Haematol..

[101] Gorski J.J., Pathak S., Panov K., Kasciukovic T., Panova T., Russell J., Zomerdijk J.C. (2007). A novel TBP-associated factor of SL1 functions in RNA polymerase I transcription. EMBO J..

[102] Chen T.H., Kambal A., Krysiak K., Walshauser M.A., Raju G., Tibbitts J.F., Walter M.J. (2011). Knockdown of Hspa9, a del(5q31.2) gene, results in a decrease in hematopoietic progenitors in mice. Blood.

[103] Agnelli L., Bicciato S., Mattioli M., Fabris S., Intini D., Verdelli D., Baldini L., Morabito F., Callea V., Lombardi L. (2005). Molecular classification of multiple myeloma: A distinct transcriptional profile characterizes patients expressing CCND1 and negative for 14q32 translocations. J. Clin. Oncol..

[104] Hebras J., Marty V., Personnaz J., Mercier P., Krogh N., Nielsen H., Aguirrebengoa M., Seitz H., Pradere J.P., Guiard B.P. (2020). Reassessment of the involvement of Snord115 in the serotonin 2c receptor pathway in a genetically relevant mouse model. Elife.

[105] Tauber H., Huttelmaier S., Kohn M. (2019). POLIII-derived non-coding RNAs acting as scaffolds and decoys. J. Mol. Cell Biol..

